# Clinical outcomes of a novel combination of lenalidomide and rituximab followed by stem cell transplantation for relapsed/refractory aggressive B-cell non-hodgkin lymphoma

**DOI:** 10.18632/oncotarget.2255

**Published:** 2014-07-25

**Authors:** Qingqing Cai, Yiming Chen, Dehui Zou, Liang Zhang, Maria Badillo, Shouhao Zhou, Elyse Lopez, Wenqi Jiang, Huiqiang Huang, Tongyu Lin, Jorge Romaguera, Michael Wang

**Affiliations:** ^1^ Department of Medical Oncology, Sun Yat-sen University Cancer Center; State Key Laboratory of Oncology in South China; and Collaborative Innovation Center for Cancer Medicine, Guangzhou, China; ^2^ Department of Lymphoma and Myeloma, The University of Texas MD Anderson Cancer Center, Houston, Texas, USA; ^3^ Lymphoma and Myeloma Center, Institute of Hematology and Blood Diseases Hospital, State Key Lab of Experimental Methods of Hematology, Chinese Academy of Medical Sciences and Peking Union of Medical College, Tianjin, China; ^4^ Department of Biostatistics, The University of Texas MD Anderson Cancer Center, Houston, Texas, USA; ^5^ Department of Stem Cell Transplantation and Cellular Therapy, The University of Texas MD Anderson Cancer Center, Houston, Texas, USA

**Keywords:** Clinical outcomes, Lenalidomide, Relapsed lymphoma, Rituximab, Stem cell transplantation

## Abstract

We retrospectively compared outcomes of patients with relapsed/refractory non-Hodgkin lymphoma (NHL) who underwent stem cell transplantation (SCT) with stable disease or better following a novel combination of lenalidomide and rituximab (LR) treatment and did not undergo SCT in a phase I/II clinical trial. We retrospectively compared outcomes of patients who underwent SCT with that of patients who had stable disease or better following LR treatment and did not undergo SCT. Twenty-two patients enrolled in LR clinical trial and undergone SCT were identified, 13 with mantle cell lymphoma (MCL) and nine with large B-cell lymphoma (LBCL). All patients who underwent SCT achieved complete response. In the MCL subset, there were no significant differences between SCT and non-SCT groups except that non-SCT patients were older and had a higher mantle-cell international prognostic index score. There was no difference between SCT-group and non-SCT-group in response duration (**P**=0.3), progression-free survival (PFS) (**P**=0.304) and overall survival (OS) (*P*=0.87). In LBCL subgroup, there were no significant differences between two groups except that non-SCT group had a higher international prognostic index score. Patients with LBCL who underwent SCT had significantly longer response duration (*P*=0.001), PFS (*P*=0.000), and OS (*P*=0.003) than the non-SCT group. The novel therapeutic combination offers a bridge to SCT in patients with relapsed/refractory aggressive B-cell NHL.

## INTRODUCTION

Approximately 40% of patients with aggressive B-cell non-Hodgkin lymphoma (B-NHL) experience disease relapse following initial immune-chemotherapy in the last years [1-5]. Results of PARMA study made high-dose chemotherapy with autologous stem cell transplantation (auto-SCT) the frontline therapy for younger and fitter patients with relapsed/refractory NHL after CHOP-like therapy.[6] Given the high rate of relapse after chemotherapy and auto-SCT, and the potential benefit of a graft-versus-lymphoma effect after allogeneic SCT (allo-SCT), however, patients with NHL are frequently considered for allo-SCT. Relapsed/refractory mantle cell lymphoma (MCL) has an especially poor outcome after auto-SCT; [7]in these patients, non-myeloablative allo-SCT has shown promising 2-year progression-free survival (PFS) rates.[8]

Chemosensitivity of disease before SCT is considered one of the most important favorable prognostic factors in SCT for NHL. However, patients with relapsed/refractory aggressive B-cell NHL often rapidly develop chemotherapy resistance. Therefore, there is a need for novel drugs and regimens to serve as effective pre-transplantation salvage regimens for these patients. Lenalidomide(Revlimid), an immunomodulatory drug, has a positive therapeutic effect in relapsed/refractory aggressive B-cell NHL, with response rates of 19-60%.[9-13] Rituximab, an anti-CD20 monoclonal antibody, can prolong overall survival (OS) and PFS in patients with CD20+ B-cell NHL when added to standard frontline therapies.[14-16] In laboratory studies *in vitro* and *in vivo*, we found that a combination of lenalidomide and rituximab (LR) provides a synergistically therapeutic effect on MCL cells by enhancing apoptosis and rituximab-dependent natural killer cell–mediated cytotoxicity.[17, 18] Furthermore, in phase I/II clinical trials, we found that a new combination of oral LR is well tolerated and effective for patients with relapsed/refractory MCL, DLBCL, or TL or chronic lymphocytic leukemia.[19-21]

But it remains unknown whether the outcomes of patients with relapsed/refractory aggressive B-cell NHL who underwent SCT after receiving LR are superior to the outcomes of patients who received LR but did not undergo SCT. In the current report, we update the outcomes of patients with relapsed/refractory aggressive B-NHL who underwent SCT after receiving LR in a phase I/II clinical trial, comparing them with the outcomes of patients in the trial who received LR but did not undergo SCT.

## RESULTS

### Characteristics of patients

The baseline demographic characteristics, treatment history, and disease status at trial enrollment for the 22 patients who underwent SCT are summarized in Table 1. Of the 13 MCL patients who underwent SCT, all had previously received a rituximab-containing regimen, and two (15%) had undergone auto-SCT before enrolling in the trial (Table 2). Four patients had a CR to LR, four had a PR, and five had stable disease. The clinical characteristics of MCL patients who underwent SCT and those who did not are shown in Table 3. There were no statistically significant differences between the two MCL groups except that the patients in the non-SCT group were older and had a higher MIPI score.

**Table 1:** Baseline demographic and clinical characteristics of all patients who underwent stem cell transplantation

Variable	All (n=22)	MCL (n=13)	LBCL* (n=9)
Age, years(range)	59.4 (46-71)	59 (46-71)	60 (49-69)
Sex, male	18(82%)	12 (92%)	6 (67%)
Time of SCT from diagnosis, months (range)	25(3-99.4)	24(3-87)	29.6(3.9-99.4)
Duration of most recent prior remission, months (range)	12.9(1-95.6)	15.8 (1-39.4)	12.9(2.6-95.6)
MIPI/IPI score (range) Prior lines of therapy, median(range)	2(0-4) 2 (1-4)	2(0-4) 2 (1-3)	1(1-4) 3 (2-4)
Number of prior therapies 1 2 3 4	6 (27%) 9 (41%) 6 (27%) 1 (5 %)	6 (46%) 5 (38%) 2 (15%) 0 (0%)	0 (0%) 4(44%) 4 (44%) 1 (11%)
Type of previous therapy			
Combination chemotherapy†	5 (23%)	2 (15%)	3 (33%)
Rituximab maintenance	1 (5%)	1 (8%)	
Rituximab combination chemotherapy§	20 (91%)	12 (92%)	8 (89%)
Bortezomib+ rituximab	1 (5%)	1 (8%)	
XRT/proton therapy	3 (14%)	1 (8%)	2 (22%)
Auto-SCT	4 (18%)	2 (15%)	2 (22%)
CCI-779	1 (5%)		1 (11%)
Disease status at enrollment			
Chemosensitive	17 (77%)	11 (85%)	6 (67%)
Chemoresistant	5(23%)	2 (15%)	3 (33%)

Abbreviations: MCL, mantle cell lymphoma; LBCL,large B-cell lymphoma; DLBCL, diffuse large B-cell lymphoma; FLG3, grade 3 follicular lymphoma; TL,transformed lymphoma; MIPI, mantle-cell international prognostic index; IPI, international prognostic index; XRT, radiation therapy; auto-SCT, autologous stem cell transplantation; CCI-779, temsirolimus,

* DLBCL (n=4); FLG3 (n=1); TL (n=4).

† Combination chemotherapy included the following: cyclophosphamide, doxorubicin, vincristine, and prednisone (CHOP); carmustine, etoposide, cytarabine, andmelphalan (BEAM); H-VAD; oretoposide, methylprednisolone, cytarabine, and cisplatin(ESHAP).

§ Rituximab combination chemotherapy included the following: CHOP; cyclophosphamide, vincristine, doxorubicin, and dexamethasone(hyper-CVAD)/high-dose methotrexate and cytarabine; ifosfamide, carboplatin, and etoposide (ICE); BEAM; gemcitabine, oxaliplatin, and ifosfamide (GIFOX);or cyclophosphamide, vincristine, and prednisolone (CVP).

Data represent number of patients (%) unless otherwise specified.

**Table 2:** Stem celltransplantation characteristics

Variable	MCL	LBCL
Transplantation before LR (all, auto-SCT; n=4)	2	2
Conditioning regimen		
R-BEAM	1	2
Busulfan+melphalan	1	0
Stem cell source Peripheral blood	2	2
Transplant after LR (n=22)	13	9
Type of transplantation	13	9
Autologous SCT	0	4
Allogeneic SCT	13	5
Interval between 1^st^ and 2^nd^ SCT, median, months(range)	28(19-36)	23(16-29)
HLA compatibility HLA incompatible	13	5
Matched sibling	3	2
Matched unrelated donor	10	3
Conditioning regimen	13	9
Myeloablative regimen	0	6
Reduced-intensity conditioning	13	3
Fludarabine based	12	3
Others	1	0
Stem cell source	13	9
Bone marrow	2	1
Peripheral blood	11	8
Acute GVHD	4	1
Grade II	2	1
Grade III/IV	2	0
Chronic GVHD	5	4
Limited	1	2
Extensive	4	2

Abbreviations: MCL, mantle cell lymphoma; LBCL, large B-cell lymphoma; LR,lenalidomide+rituximab; SCT, stem cell transplantation; R-BEAM, rituximab,carmustine,etoposide,cytarabine,andmelphalan; HLA, human leukocyte antigen; GVHD,graft-versus-host disease.

Data represent number of patients (%) unless otherwise specified.

**Table 3:** Baseline demographic and clinical characteristics of MCL patients by SCT status

Factor	SCT (n=13)	Non-SCT (n=39)	Pvalue
Age, years (range)	59(46-71)	71(50-85)	0.001
Sex			
Male	12 (92%)	35 (90%)	1.000
Female	1 (8%)	4 (10%)	
Time from diagnosis, months (range)	24 (3-87)	32(3-95)	0.106
Bone marrow involvement at study entry, median (range)	6 (46%)	18(46%)	1.000
Duration of last remission, months (range)	14 (0-39)	12(0-49)	0.751
Previous lines of therapy, median (range)	2 (1-3)	2 (1-4)	0.173
Mantle-cell international prognostic index score, median(range)	2 (0-4)	3 (1-7)	0.006

Abbreviations: MCL, mantle-cell lymphoma; SCT, stem cell transplantation. Data represent median (range) or number(%).

All nine patients with LBCL had previously received a rituximab-containing regimen. Two patients (22%) had undergone SCT before enrolling in the trials (Table2). Six patients had a CR to LR and three had a PR. The clinical characteristics of LBCL patients who underwent SCT and those who did not are shown in Table 4. There were no statistically significant differences between the two groups except that the non-SCT group had a higher IPI score.

**Table 4:** Baseline clinical characteristics of LBCL patients by SCT status

Variable	SCT (n=9)	Non-SCT (n=36)	P value
Age, years (range)	60(49-69)	69.5(24-85)	0.055
Sex			1.000
Male	6 (67%)	22 (61%)	
Female	3 (33%)	14 (39%)	
Pathologic type			0.075
DLBCL	4 (44%)	28(78%)	
FLG3	1 (11%)	3 (8%)	
TL	4 (44%)	5 (14%)	
Stage 4 at diagnosis, number	3 (33%)	18(50%)	0.469
Time from diagnosis, months (range)	29.6(4-99)	21.5(4-98)	0.580
Prior lines of therapy, median (range)	3 (2-4)	3 (1-4)	0.315
International prognostic index score, median (range)	1 (1-4)	3 (1-5)	0.008

Abbreviations: LBCL, large B-cell lymphoma; SCT, stem cell transplantation; DLBCL, diffuse large B-cell lymphoma; FLG3,grade 3 follicular lymphoma; TL,transformedlymphoma.

### Stem cell transplantation

Of the 13 MCL patients who underwent SCT, the median interval from date of best response to LR to date of SCT was 4 months (range, 1-11months). All of the patients achieved CR following SCT. After a median follow-up interval of 24months from SCT (range, 13-90 months), four of the 13 patients are alive. One (8%) patient died of progressive disease, and eight (62%) died of complications following post-LR allo-SCT, including GVHD, pneumonia, and sepsis. The NRM rate was 62%.

Of the nine LBCL patients who underwent SCT, the median interval from date of best response to LR to date of SCT was 3 months (range, 1-14 months). Four patients who had a CR following LR underwent auto-SCT. The other five patients, three of whom had a PR following LR and the other two disease relapse after auto-SCT, underwent allo-SCT. All of the patients achieved CR following post-LR SCT. After a median follow-up interval of 38 months from SCT (range, 6-60 months), seven of the nine patients are alive (DLBCL, 3; TL, 3; FLG3, 1). One(11%) patient, who had DLBCL, died of GVHD following allo-SCT; another (11%), who had FLG3, died of liver failure following auto-SCT. The NRM rate was 22%.

### Outcomes

**Figure 1:**
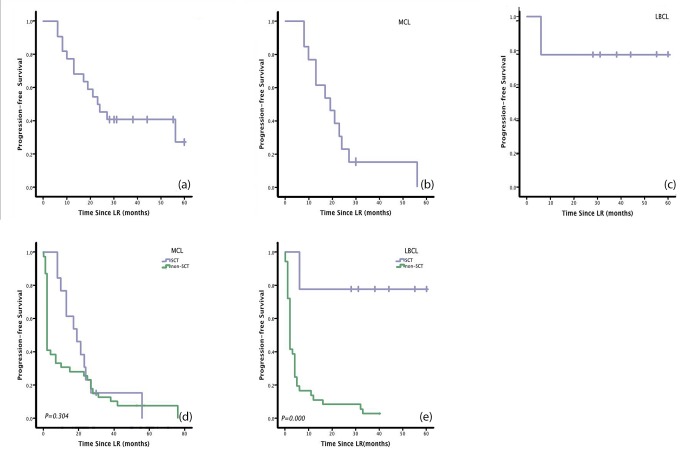
Patients with large B cell lymphoma (LBCL) who underwent stem cell transplantation (SCT) had significantly longer Progression-free survival (PFS)than patients who received lenalidomide + rituximab therapy (LR) without SCT Patients with mantle cell lymphoma (MCL) who underwent SCT, on the other hand, did not show any benefit in PFS. (a) PFS after LR for all patients enrolled in the phase I/II clinical trial.(b) PFS for all MCL patients enrolled in the trial.(c) PFS for all LBCL patients enrolled in the trial.(d) PFS in MCL patients who underwent SCT and those who did not. (e) PFS in LBCL patients who underwent SCT and those who did not.

**Figure 2:**
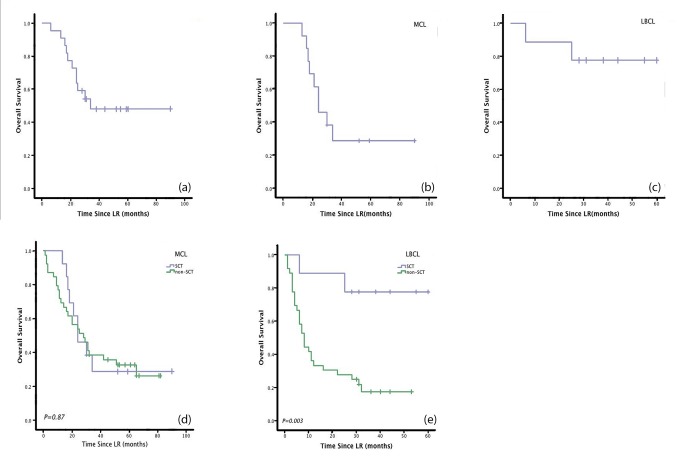
Patients with LBCL who underwent SCT had significantly longer OS than patients who received LR without SCT Otherwise, patients with MCL who underwent SCT did not show any benefit in OS.(a) Overall survival (OS) after lenalidomide± rituximab therapy (LR) for all patients enrolled in the phase I/II clinical trial. (b) OS for MCL patients enrolled in the trial.(c) OS for LBCL patients enrolled in the trial.(d) OS in MCL patients who underwent SCT and those who did not. (e) OS in LBCL patients who underwent SCT and those who did not.

The median PFS of the SCT patients in this retrospective study was 23 months. Estimated 1-year, 3-year, and 5-year PFS rates were 77.3%, 40.9%, and 27.3%, respectively (Figure 1a). The median OS was 34 months. Estimated 1-year, 3-year, and 5-year OS rates were95.5%, 48.1%, and 48.1%, respectively (Figure 2a).

**Figure 3:**
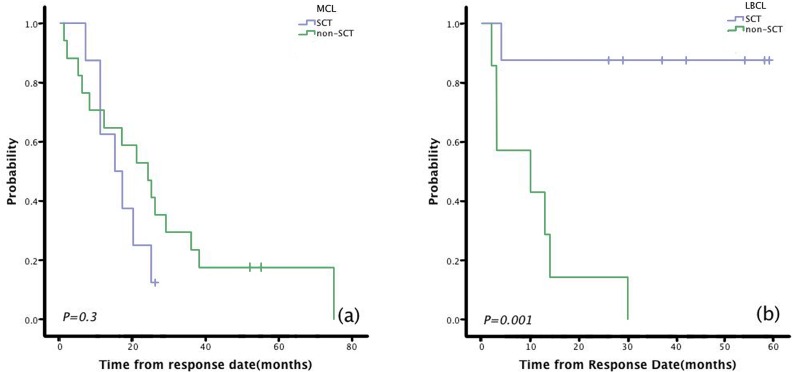
Patients with LBCL who underwent SCT had significantly longer response duration than patients who received LR without SCT However, patients with MCL who underwent SCTdid not show any benefit in response duration. (a) Durations of response to lenalidomide + rituximab (LR) in MCL patients who underwent SCT and those who did not. (b)Durations of response to LR in LBCL patients who underwent SCT and those who did not.

Among the 13 MCL patients who underwent SCT, the median PFS was 19 months. Estimated 1-year, 3-year, and 5-year PFS rates were 76.9%, 15.4%, and 0%, respectively (Figure 1b). The median OS was 24 months. Estimated 1-year, 3-year, and 5-year OS rates were 92.3%, 28.8%, and 28.8%, respectively (Figure 2b). Compared with non-SCT patients, patients with MCL who underwent SCT did not have longer response duration (15 vs 24 months, *P*=0.3;Figure 3a), PFS (19 vs 2 months, *P*=0.304; Figure 1d),or OS (24 vs 28 months, *P*=0.87; Figure 2d).

Among the nine LBCL patients who underwent SCT, the median PFS was not reached. Estimated 1-year, 3-year, and 5-year PFS rates were 77.8%, 77.8%, and 77.8%, respectively (Figure 1c). The median OS was not reached. Estimated 1-year, 3-year, and 5-year OS rates were 88.9%, 77.8%, and 77.8%, respectively (Figure 2c). Patients with LBCL who underwent SCT had significantly longer response duration(NR vs 10 months, *P*=0.001; Figure 3b), PFS (NR vs 2 months, *P*=0.000;Figure 1e), and OS (NR vs 8 months, *P*=0.003; Figure 2e) than patients who did not undergo SCT.

### Efficacy of stem cell collection in patients who underwent autologous hematopoietic stem cell transplantation

Four of the study patients, two with DLBCL and two with TL, underwent auto-SCT. All patients received rituximab+ifosfamide+etoposide combined with granulocyte colony-stimulating factor for autologous stem cell mobilization before the LR treatment. CD34+ cell collection (>2×10^6^/kg CD34+cells) was successful for all four patients (100%) within a median of 3 apheresis days (range, 2-5). Median peripheral CD34+ cell count for the four patients was 5.02 cells/μl (range, 4.58-5.5). Hematopoietic recovery after auto-SCT occurred at the expected time (median intervals to neutrophil and platelet engraftment were 10 days and 11 days, respectively).

### Post-progression regimens

Of the 13 MCL patients who underwent SCT, nine (69%) received no further treatment after SCT. The other four (31%) received a cytotoxic chemotherapy regimen that included fractionated cyclophosphamide and high-dose cytarabine with etoposide, cisplatin, and methylprednisolone (ESHAP). Two patients (15%) received a combination of rituximab with bortezomib. Two patients (15%) received at least one novel agent, including a JAK2 inhibitor, AKT inhibitor MK2206, or BTK inhibitor ibrutinib.

Of the 39 non-SCT MCL patients, 17 (44%) received no further treatment after LR. Eleven patients received one additional chemotherapy regimen, three patients received two, three patients received three, three patients received five, and one patient received six. Seven of the patients (18%) received acytotoxic chemotherapy regimen, including hyperfractionated cyclophosphamide, vincristine, doxorubicin, and dexamethasone(hyper-CVAD)±rituximab alternating with high-dose methotrexate and cytarabine±rituximab; rituximab combined with fractionated cyclophosphamide;rituximab combined withifosfamide, carboplatin, and etoposide(R-ICE); rituximab combined with ESHAP (R-ESHAP); orrituximab combined withfludarabine, cyclophosphamide, and mitoxantrone(R-FCM). Eleven patients (28%) receivedrituximab±bortezomib. Six patients (15%) received rituximab±bendamustin. One patient (3%) received radioimmunotherapy (ibritumomabtiuxetan). Twelve patients (30%) received at least one novel agent, including a JAK2 inhibitor, BTK inhibitor ibrutinib, cyclin-dependent kinase inhibitor flavopiridol, mTOR inhibitor temsirolimus, or anti-CD19 antibody.

None of the nine LBCL patients who underwent SCT received further treatment after SCT. Of the 36 LBCL patients who did not undergo SCT, nine (25%) received no further treatment after LR. Seventeen patients received one additional chemotherapy regimen, five patients received two, one patient received three, one patient received four, and eight patients received one. Nineteen (53%) received a cytotoxic chemotherapy regimen, including hyper-CVAD±rituximab; rituximab combined with fractionated cyclophosphamide; rituximab combined with gemcitabine and oxaliplatin (R-Gemox); R-ESHAP; rituximab combined withifosfamide, mitoxantrone, etoposide, and prednisone (R-MINE); rituximab combined with paclitaxel and topotecan (R-TT);etoposide, vincristine, doxorubicin, cyclophosphamide, and prednisone (EPOCH); orrituximab combined with cisplatin, cytarabine, and dexamethasone (R-DHAP). Five patients (14%) received rituximab ±bendamustin. One patient (3%) received radioimmunotherapy (ibritumomabtiuxetan). Eight (22%) patients received at least one novel agent, including a JAK2 inhibitor, temsirolimus, anti-TRAIL antibody conatumumab(AMG655), histone deacetylase inhibitor vorinostat, MK2206, or heat shock protein-90 inhibitor AUY-992.

**Table 5:** Clinical outcomes stratified by disease subtype

Outcome	All (n=22)	MCL (n=13)	LBCL* (n=9)
Time to first response, median, months(range)	2 (1-4)	2(2-4)	2(1-2)
Time to best response,median, months (range)	2 (1-10)	2(2-10)	2(1-3)

Complete remission in response to SCT	22(100%)	13(100%)	9(100%)
Progression-free survival, median, months (range)	23(14-32)	19(10-28)	NR
Overall survival, median, months (range)	44(NR)	24(13-35)	NR

Abbreviations:MCL, mantle cell lymphoma; LBCL,large B-cell lymphoma; DLBCL,diffuse large B-cell lymphoma; FLG3,grade 3 follicular lymphoma;TL,transformed lymphoma; SCT, stem cell transplantation; NR, not reached.

Data represent number of patients (%) unless otherwise specified.

* LBCL includes DLBCL,FLG3, and TL.

## DISCUSSION

In this retrospective analysis, we assessed the clinical activity and long-term outcome of the novel combination of LR before SCT in patients with relapsed/refractory aggressive B-cell NHL. We found that patients with LBCL who underwent SCT had significantly longer response duration, PFS, and OS than patients who received LR without SCT. Patients with MCL who underwent SCT, on the other hand, did not show any benefit in response duration or survival. The novel combination of LR provides a bridge to SCT in our heavily pretreated patient population.

In relapsed LBCL, auto-SCT is the treatment of choice, resulting in long-term disease control in 40-50% of patients with chemosensitive disease but in only 10-20% patients with refractory disease.[22-26] Several recent studies have demonstrated that allo-SCT, made safer by improvements in supportive treatment and increased use of RIC regimens, may be used successfully as salvage treatment for patients with relapsed/refractory DLBCL, particularly those whose disease progresses after auto-SCT.[24, 27-31]. In our study, four LBCL patients underwent auto-SCT, all of whom had a CR following LR. Another five patients underwent allo-SCT, three who had a PR following LR and two whose disease relapsed after auto-SCT. Of patients with LBCL, those who underwent SCT had significantly longer response duration, PFS, and OS than patients who did not undergo SCT, although many non-SCT LBCL patients received novel therapies following LR. The 5-year PFS and OS of 77.8% after SCT suggest a benefit with the LR combination in patients with heavily pretreated relapsed/refractory aggressive LBCL. Further studies of more patients with LBCL who are treated with LR followed by SCT will be necessary to consolidate our promising results.

Patients with MCL who underwent SCT did not have longer response duration, PFS, or OS than the patients with MCL who did not undergo SCT, although the patients in the non-SCT group were older and had a higher MIPI than the SCT group. Nine of the 13 patients with MCL who underwent SCT died, eight (89%) of them from complications related to allo-SCT. The primary reasons for this relatively high mortality rate may be heavy pretreatment, poor performance status, and older age in the group who underwent allo-SCT. While allo-SCT using RIC has been evaluated as a consolidation strategy for patients whose disease is in remission following treatment for relapsed/refractory MCL,[8, 32, 33] further studies are needed to decrease rates of complications such as infection and/or GVHD in these patients. However, there are still many questions regarding the optimal timing or modality of SCT in MCL.[34] Allo-SCT may represent a graft-versus-lymphoma effect. However, the potential benefit of RIC–allo-SCT in terms of long-term disease control was negated by higher NRM rates after allo-SCT. Furthermore, novel agents offer a promising alternative to SCT for MCL patients with progression of disease following LR, such as the BTK inhibitor ibrutinib. Ibrutinib yields response rates of approximately 70%[35] in relapsed MCL, a finding that may change MCL treatment paradigms.

In the current era of rituximab during frontline therapy for aggressive B-cell NHL, the outcome of salvage treatments is very poor for patients with relapsed/refractory NHL. Loss of CD20 expression may lead to rituximab resistance at the time of relapse.[36] Nevertheless, in our heavily pretreated patient population, 100% of whom had previously received rituximab-containing therapy and 23% of whom were shown to have chemotherapy-refractory disease immediately before study entry, and which included four patients who had previously undergone SCT, the LR combination provided a relatively high response rate. Although we cannot directly compare these outcomes with those of other salvage regimens (many of which include conventional cytotoxic drugs), the ORRs of 57% in MCL[19] and 33% in LBCL[37] and the 3-year PFS ratesafter SCT of 15.4% in MCL and 77.8% in LBCL suggest a benefit with the LR + SCT combination in patients with relapsed/refractory aggressive B-cell NHL.

In a collaborative trial in relapsed aggressive lymphoma study, ORR for salvage chemotherapy (R-ICE or R-DHAP) was 51%.[23] The response and survival in our retrospective study were comparable to those of that clinical trial. The results of our study may be due in part to selection bias. But the LR combination is still a promising salvage therapy in heavily pretreated patients with relapsed/refractory aggressive B-NHL for whom SCT is indicated. Furthermore, the toxic effects of the LR combination are more predictable and manageable than those of conventional cytotoxics alvage regimens.[19, 37] Most importantly, LR treatment does not affect the mobilization efficacy of autologous stem cells in LBCL patients.

Although 23% of the patients maintained stable disease after LR, all of the patients achieved CR following SCT and approximately 50% achieved long-term OS after SCT. NHL that is only stable after chemotherapy is often considered to be “chemotherapy refractory” and is usually excluded from SCT trials. These studies suggest that patients with this type of disease might have a response to LR and benefit from reduced-intensity allo-SCT.[38, 39]

It is difficult to make a definitive judgment from our results alone, because this is a retrospective analysis of small cohort and the patients’ clinical characteristics are quite heterogeneous. A randomized, prospective study of a larger population is recommended to define the role of LR in the treatment of patients with relapsed/refractory aggressive B-NHL before SCT.

In summary, patients with LBCL in this retrospective study who underwent SCT experienced longer response duration, PFS, and OS than non-SCT patients. The novel combination of LR offers a bridge to SCT in patients with relapsed/refractory aggressive B-cell NHL.

## METHODS

### Study design, treatment, and patients

Between February 10, 2006, and July 30, 2009, 52 patients with relapsed/refractory MCL were enrolled in a phase I/II clinical trial of LR [19] at The University of Texas MD Anderson Cancer Center, and 45 patients with relapsed/refractory DLBCL, FLG3, or TL were enrolled in the phase II portion of the study.[20] Sixty-three patients achieved stable disease or better on the study protocol. Among these patients, 22 underwent SCT after receiving LR, 13 with MCL and nine with large B-cell lymphoma (LBCL).

Patients in the trial received 20 mg oral lenalidomide once daily on days 1-21 of each 28-day cycle and 375mg/m^2^of intravenous rituximab once a week for 4 weeks only during cycle 1,beginning on day 1, as described previously; the only exceptions were two MCL patients from phase I, one of whom received 10mg and the other 15mg oral lenalidomide once daily on days 1-21.[19, 20] Patients were treated until disease progression, SCT, or withdrawal for toxicity. The lenalidomide dose was reduced from 20mg to 15mg, 10mg, and 5mg in a progressive fashion if a patient experienced grade 3 or 4 non-hematological toxic effects or grade 4 hematological toxic effects in the phase II study. All patients signed an informed consent and the trial was approved by the institutional review board of MD Anderson Cancer Center. This retrospective analysis was also approved by the institutional review board. Informed consent was obtained from patients.

### Stem cell transplantation

Of the 22 patients who underwent SCT, 18 underwent allo-SCT and four auto-SCT following the clinical trial. All of the MCL patients underwent allo-SCT with a reduced-intensity conditioning (RIC) regimen. The preparative regimens were fludarabine + cyclophosphamide + rituximab (n=1), fludarabine + cyclophosphamide + rituximab + alemtuzumab (n=3), fludarabine + cyclophosphamide + rituximab + alemtuzumab with low-dose total body irradiation (n=3), fludarabine + cyclophosphamide + rituximab + ibritumomabtiuxetan(n=3), fludarabine + bendamustine (n=1), fludarabine + busulfan(n=1), and busulfan+melphalan+alemtuzumab(n=1). Ten patients received stem cells from a matched unrelated donor and three from a matched sibling donor.

Of the LBCL patients, four underwent auto-SCT and five allo-SCT. Of the five allo-SCT patients, two were given a myeloablative conditioning regimen and the other three RIC regimen. The preparative regimens were fludarabine + cyclophosphamide + rituximab (n=2), fludarabine + bendamustine + rituximab (n=1), cyclophosphamide + total body irradiation (TBI) (n=1) and rituximab, carmustine, etoposide, cytarabine, and melphalan (R-BEAM) + bortezomib (n=1). Three received stem cells from a matched unrelated donor and two from a matched sibling donor. All of the four patients who underwent auto-SCT received R-BEAM.

### Data analysis

Response to SCT was assessed according to guidelines developed by the International Workshop on Lymphoma Response (Criteria #2.0).[40] Restaging was done after every 2 cycles and was based on computed tomography (CT) scans and bone marrow biopsy findings. All original radiological evaluations (baseline and follow-up) were reassessed for this study by a designated radiologist. The same radiologist reviewed the baseline and follow-up CT scans.

The primary efficacy endpoints of this study were PFS and OS. The secondary efficacy endpoints were overall response rate (ORR), complete response (CR), or partial response (PR) to post-LR SCT. OS was defined as the time from study entry to the date of death or the date of the last follow-up. PFS was defined as the time from study entry to the date of disease progression or death or the date of the last follow-up. For the response duration, responders who went off study for any reason other than disease progression or death were censored on the last CT scan date, either before going off study or within 2 weeks of being off study. Responders who did not experience disease progression but died of another reason were censored at the date of death. Responders who were still actively on study were censored at the survival date (i.e., date of last follow-up) or the last CT scan date, whichever was later. Non-relapse mortality(NRM; time to death without relapse/recurrence)and incidence of graft-versus-host disease (GVHD) were also analyzed.

Continuous variables were compared by the Mann-Whitney U test. Categorical characteristics were compared by chi-square test or Fisher exact test. The Kaplan-Meier method was used to determine the probability of OS and PFS as a function of time. A log-rank test was used to determine significance of differences among Kaplan-Meier curves for OS or PFS. *P* values < 0.05 were considered statistically significant. All statistical calculations were done with SPSS 20.0 software (SPSS Inc., Chicago, IL, USA).
